# Negative-Ion Formation upon Soft X-ray Photoexcitation of 2-Propanol

**DOI:** 10.1021/acs.jpca.4c08226

**Published:** 2025-02-27

**Authors:** Antti Kivimäki, Eetu Pelimanni, Kirill Chernenko, Abdul Rahman Abid, Christian Stråhlman

**Affiliations:** † MAX IV Laboratory, Lund University, 22100 Lund, Sweden; ‡ Nano and Molecular Systems Research Unit, University of Oulu, 90570 Oulu, Finland; § Chemical Sciences and Engineering Division, Argonne National Laboratory, Lemont, Illinois 60439, United States; ∥ Molecular and Condensed Matter Physics, Uppsala University, 75120 Uppsala, Sweden; ⊥ Department of Materials Science and Applied Mathematics, Malmö University, 20506 Malmö, Sweden

## Abstract

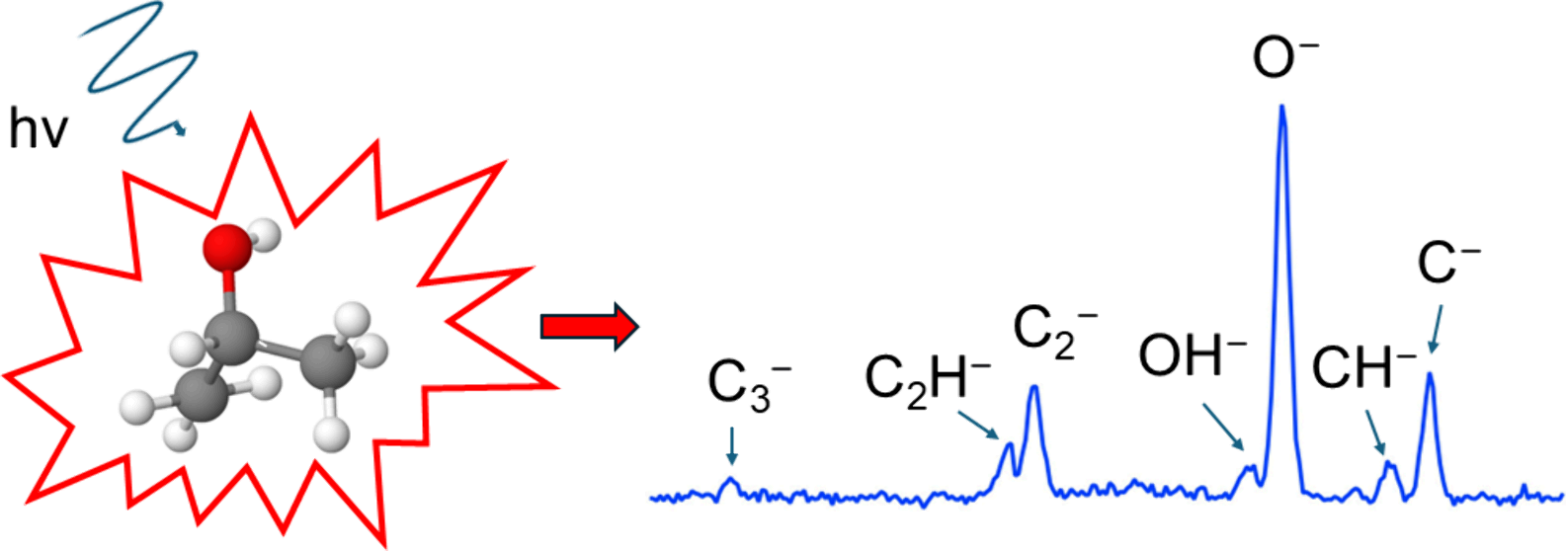

This study investigates the formation of negative ion fragments from gas-phase 2-propanol molecules after interaction with soft X-rays near the O K-edge. The experiment was performed by detecting negative and positive ions in coincidence with time-of-flight spectrometry. The analysis of two- and three-ion coincidence data revealed that nine different anions were produced: H^–^, C^–^, CH^–^, CH_2_^–^, O^–^, OH^–^, C_2_^–^, C_2_H^–^, and C_3_^–^. For all anions, the most common three-ion events were those involving two protons. The results highlight the sensitivity of negative-ion/positive-ion coincidence spectroscopy and provide new insight into the fragmentation processes of organic molecules under soft X-ray excitation.

## I Introduction

The study of anions and their formation conditions spans multiple scientific disciplines due to their significant roles in foundational chemical, physical and biological processes. To thoroughly understand the origins and role of anions in complex molecular interaction chains, e.g., in the context of astrochemistry^1^ or atmospheric chemistry,^2^ it is necessary to explore the mechanisms that can lead to anion production at the fundamental level. Anion formation from neutral precursor molecules often occurs via electron attachment,^1^ but anions can also be generated through molecular fragmentation via photoabsorption. This was first observed under vacuum ultraviolet (VUV) radiation for TlI salt molecules, which produced Tl^+^ and I^–^ ions.^3^ This phenomenon, ion-pair formation, can be described for a generic molecule AB by the reaction: AB + *h*ν → AB* → A^–^ + B^+^, where the (Rydberg) excited molecule AB* predissociates into an ion pair. In diatomic molecules, ion-pair formation could be studied simply by detecting a mass-selected anion as a function of the photon energy,^4^ as the positive fragment became known by the same selection. Dujardin et al.^5^ observed that fragment anions were formed near the direct-double-ionization and S 2p core-ionization thresholds of the SO_2_ molecule, attributing this at least partly to the existence of superexcited states of the singly charged parent ion (superexcited states are lying above the ionization energy of the species—in this case, above the double-ionization-threshold energy). Subsequent X-ray studies recorded yields of negative ions at the core edges of several simple molecules such as methanol,^6^ carbon dioxide,^7^ and water.^8^ Anion yields increase particularly at core-to-valence and core-to-Rydberg resonances, which are located below core-level ionization potentials in absorption spectra, but anions can also be formed in the core level photoionization continuum regime.

If a polyatomic molecule has several dissociation channels involving the same anion, coincidence detection between negative and positive ions can distinguish them. A principally straightforward way to perform such experiments is to use two ion time-of-flight (TOF) spectrometers: one for positive ions and the other for negative ions.^9,10^ If velocity imaging capability is included, information on the kinetic energies of and the angular differences between the released ions may also be obtained.^11^ The negative-ion/positive-ion coincidence (NIPICO) instrument used in the present work is likewise based on two ion TOF spectrometers.^12^

It is interesting to consider how the variety of different anionic fragments produced upon core-excitation changes with increasing system size, and to apply the NIPICO technique to larger polyatomic molecules. Let us consider simple alcohols for example. For methanol (CH_3_OH), Stolte et al.^6^ observed the production of H^–^, O^–^, and OH^–^ with anion yield spectroscopy, and in a later study using NIPICO spectroscopy C^–^ and CH^–^ were also detected.^13^ NIPICO experiments were recently performed at the O 1s edge of ethanol (CH_3_CH_2_OH) using the present experimental setup, and their analysis revealed the same five anions as in methanol and additionally C_2_^–^ and C_2_H^–^.^14^ In this work, we study anion formation in 2-propanol (iso-propanol, CH_3_–CHOH–CH_3_) at the O 1s edge, and observe a multitude of dissociation channels involving up to nine different anions.

## II Experimental Section

The experiments were performed at the FinEstBeAMS beamline^15,16^ at the MAX IV Laboratory (Lund, Sweden). The experimental setup consisted of two ion time-of-flight (TOF) spectrometers mounted opposite one another. The positive ion TOF spectrometer was borrowed from the gas-phase end station,^17^ where it is regularly used for photoelectron-photoion coincidence (PEPICO) spectroscopy. It has a position-sensitive detector (from RoentDek Handels GmbH) equipped with 80 mm microchannel plates (MCP) and a Hex-anode delay-line setup. The TOF spectrometer for negative ions was designed and constructed at the MAX IV Laboratory. It was initially used with another ion TOF spectrometer at Elettra (Trieste, Italy),^12^ and later returned to the MAX IV Laboratory. The current setup is based on the same operation principle as the one described in ref (12). Both spectrometers shared a common extraction region for positive and negative ions. Measurements were performed using a constant extraction field under normal multibunch operation of the MAX IV 1.5-GeV storage ring. An external magnetic field, created by placing permanent magnets outside the vacuum tube of the negative ion TOF spectrometer, reduced the number of electrons reaching the detector. This tandem TOF setup allows the collection of various spectra, including total (positive) ion yields, partial electron yields, PEPICO spectra (without analyzing the kinetic energies of electrons), and negative-ion positive-ion coincidence (NIPICO) spectra.

An event was considered a coincidence when both the ion TOF spectrometers detected particles within a selectable brief time (e.g., 20 μs) from each other. Signals from the negative particle detector were treated as START signals and those from the positive particle detector as STOP signals, effectively yielding an arrival-time-difference (ATD) spectrum: ATD = TOF(positive ion) – TOF(negative ion or electron). As the flight times of given positive and negative ions are well-defined in TOF spectrometers, the differences between their flight times are also well-defined, meaning that each negative-ion/positive-ion pair creates a distinct peak in an ATD spectrum. Coincidence events where a positive ion arrived before a negative ion were also registered, resulting in a negative ATD. The multihit capability of the positive ion detector allowed us to analyze events where two positive ions were created together with one negative ion. Negative-ion/positive-ion/positive-ion coincidences (NIPIPICOs) could therefore be observed.

PEPICO spectra were collected with the same setup by allowing more electrons to hit the negative particle detector, achieved by removing some of the permanent magnets. (We did not remove all the magnets because that would have made it more difficult to reset the experimental conditions for NIPICO experiments.) Since far more electrons than negative ions were produced in these conditions, the relative intensities of the NIPICO peaks decreased drastically and they practically disappeared from the spectrum. The kinetic energies of the electrons were not resolved in these PEPICO measurements.

The analysis of the PEPICO and PEPIPICO spectra revealed the presence of N_2_ and Ar in the vacuum chamber. The latter can be explained by a misfunctioning leak valve between a gas reservoir and the vacuum chamber that was letting some gas through. Since the NIPICO experiments were conducted during the same beamtime as the PEPICO measurements, the sample vapor was not pure 2-propanol. However, these impurities do not invalidate our observations, noting also that N^–^ is very unlikely to be observed.^18^

## III Results and Discussion

### III.1 O 1s Excitation Spectrum

**Figure 1:**
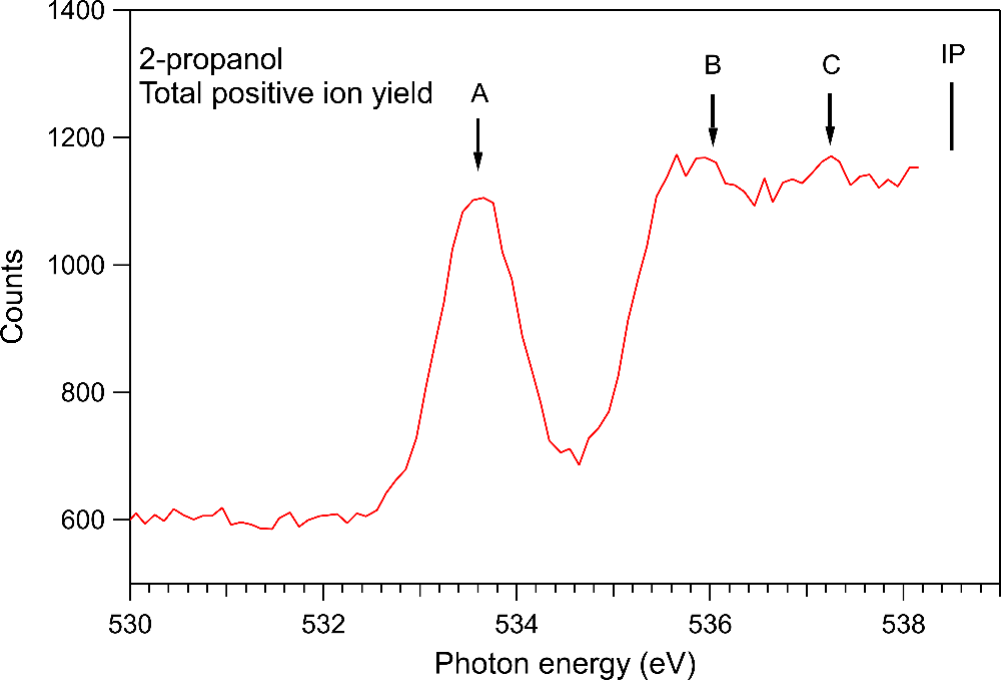
Total positive ion yield of 2-propanol measured below the O 1s ionization potential (IP). The arrows indicate photon energies that were used to collect the NIPICO spectra of 2-propanol.

During the beamtime when the NIPICO measurements were performed, the optical elements of the monochromator were strongly contaminated with carbon, making absorption-type measurements impossible at the C 1s edge and difficult at the O 1s edge. The total positive ion yield at the O K-edge of 2-propanol only showed one distinct peak at around 534 eV. The optics were subsequently cleaned and the positive ion yield spectrum of 2-propanol at the O K-edge was remeasured in better conditions. This result is shown in Figure 1 after normalization to the photodiode current measured simultaneously. The photon energy scale was calibrated according to the total ion yield spectrum of Thomas et al.,^19^ which placed the first O 1s resonance of 2-propanol at 533.6 eV. In the earlier beamtime, the other photon energies for NIPICO measurements were chosen by using the energy differences between the O 1s excitations of 2-propanol as observed by Thomas et al.^19^ These energies are indicated by arrows in Figure 1. Our total ion yield spectrum is like the one measured by Thomas et al. These authors assigned the features A, B, and C to the excitations of O 1s electrons to the σ*(O–H), 3p, and σ*(C–O) orbitals, respectively; their assignment was based on the core excitation spectrum of 1-propanol that had been interpreted by Ishii and Hitchcock.^20^ Note that the positive ion yield is rather high before the O 1s excitations, which is mostly due to C 1s photoionization processes along with a smaller contribution from valence photoionization processes. The signal levels observed in our spectrum before and at the resonances agree with the results of Thomas et al.^19^ and qualitatively also with the calculated atomic photoionization cross sections.^21^

### III.2 NIPICO and PEPICO Spectra

**Figure 2:**
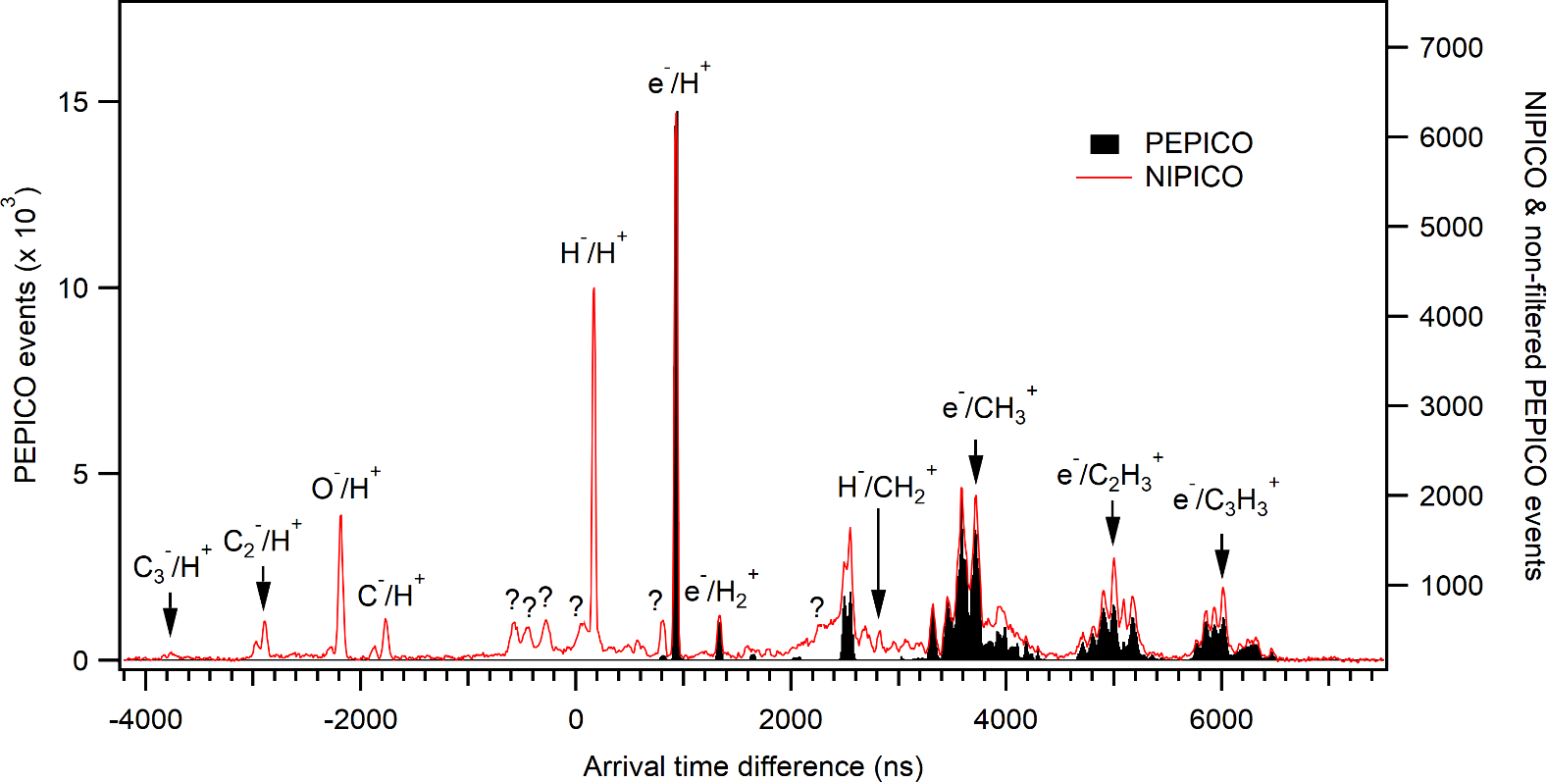
Arrival time difference spectra of 2-propanol measured using the NIPICO and PEPICO settings at the photon energy of 536.1 eV. The acquisition time was 8 h 20 min for the NIPICO spectrum. The PEPICO spectrum was measured for 1000 s, and the electron count rate was limited to about 470 Hz by the exit slit size of the monochromator and the magnets placed outside the vacuum chamber.

Figure 2 shows the NIPICO and PEPICO spectra of 2-propanol measured at the photon energy of 536.1 eV (feature B in Figure 1). The intensities are scaled to the same heights for the most intense peak in both spectra, which corresponds to e^–^/H^+^ coincidences. Other peaks originating from coincidences between electrons and positive ions appear at larger ATDs than the e^–^/H^+^ peak. Some of them are labeled in Figure 2. Note that the atomic mass numbers of the positive ions change by 1 for the adjacent peaks within three groups of peaks between 3300 and 6200 ns. Thus, for example, in the peak group located around ATD = 5000 ns, the following positive ions in the order of increasing ATD are observed: C_2_^+^ (at 4700 ns), C_2_H^+^, C_2_H_2_^+^, C_2_H_3_^+^, C_2_H_4_^+^ or CO^+^, and C_2_H_5_^+^ or COH^+^ (at 5170 ns). The dual-peak PEPICO feature at around 2500 ns likely originates from e^–^/N^2+^ coincidences. Consequently, the PEPICO signal around 3600 ns is probably due to the sum of e^–^/N^+^ and e^–^/CH_2_^+^ coincidences. Residual water in the vacuum chamber (in the partial pressure range of 10^–8^ mbar) may have contributed to the signal around 3800 to 4000 ns (O^+^, OH^+^, and H_2_O^+^).

Peaks arising from coincidences between negative ions and positive ions can appear at both negative and positive ATD values. NIPICO peaks at negative ATDs generally involve an anion heavier than the cation measured in coincidence. The NIPICO peaks with the most negative ATD values involve the heaviest anions and the lightest cation, H^+^. The NIPICO peaks where H^+^ was observed with different anions allow us to construct an equation for the flight times of the anions as a function of their mass-to-charge ratio. Consequently, the ATD of any negative-ion/positive-ion coincidence can be predicted accurately (within ±10 ns of the observed peak positions). The NIPICO spectrum in Figure 2 reveals that the following anions were observed in coincidence with H^+^: H^–^, C^–^, CH^–^, O^–^, OH^–^, C_2_^–^, C_2_H^–^, and C_3_^–^.

Coincidences between negative ions and other, heavier, positive ions are also seen in Figure 2, especially at positive ATDs. However, due to their weaker intensities and overlap with PEPICO features, we discuss these assignments in more detail in the next section in connection with the NIPIPICO map, from which they are much easier to resolve and identify. We note, however, that for a given series, such as H^–^/PI, where the negative ion is the same and the positive ion changes, the expected positions of the coincidence peaks in Figure 2 can be found easily after noticing that a NI/PI coincidence peak always appears at the same distance from the corresponding e^–^/PI peak. This distance is the flight time difference between the electrons and the negative ions, and it can be seen in Figure 2, for example, between the H^–^/H^+^ and e^–^/H^+^ peaks. Accordingly, the peaks with ATD values of 2650–3200 ns in Figure 2 can be attributed to the H^–^/PI coincidences, where PI= CH^+^, CH_2_^+^, CH_3_^+^, O^+^, and OH^+^. The missing first member of the series, H^–^/C^+^, is predicted to be at 2540 ns, overlapping with the two-peaked PEPICO feature due to e^–^/N^2+^ coincidences.

**Figure 3:**
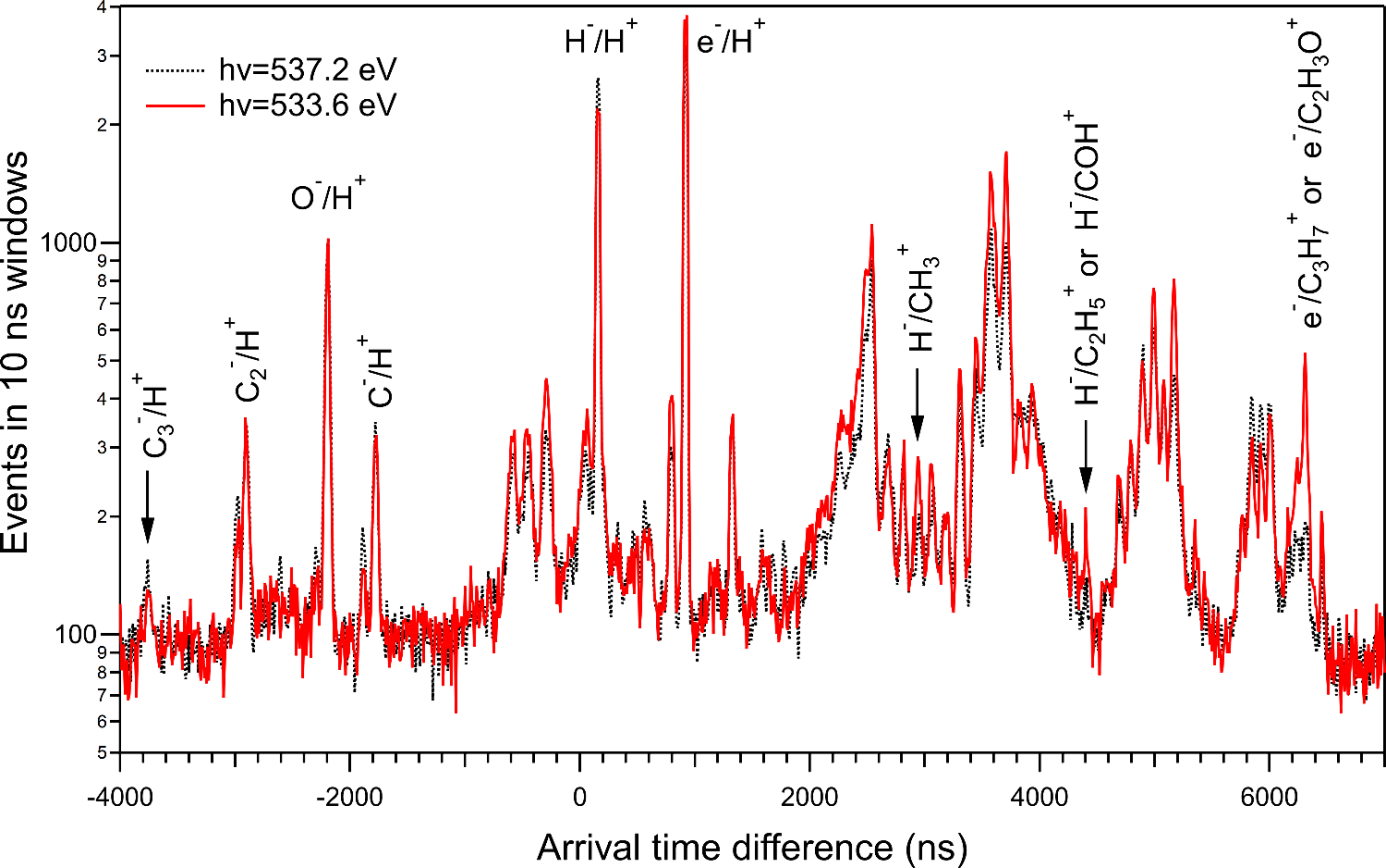
Arrival time difference spectra of 2-propanol measured using the NIPICO settings at the photon energies 533.6 and 537.2 eV. The *y*-axis is logarithmic to enhance differences in low-intensity peaks.

The NIPICO spectra measured at features A, B, and C (see Figure 1) are slightly different. Figure 3 shows a comparison of the relative intensities of the NIPICO peaks for photon energies 533.6 and 537.2 eV (features A and C in Figure 1) by scaling the O^–^/H^+^ peaks at the same height. The spectrum measured at 536.1 eV (Figure 2) is not shown for clarity, but it resembles more the 537.2 eV spectrum than the 533.6 eV spectrum. Although the statistics are rather low, we can observe that the relative intensities of some NI/H^+^ peaks decreased at 533.6 eV photon energy. This is most certain for the H^–^/H^+^ and CH^–^/H^+^ peaks (the latter is at −1900 ns). In contrast, some small H^–^/PI peaks, most notably for PI = CH_3_^+^ (at 2940 ns) and C_2_H_5_^+^ or COH^+^ (at 4400 ns), increased in relative intensity at 533.6 eV. In addition, a PEPICO peak at 6300 ns clearly became more intense at 533.6 eV photon energy. It could be assigned to either C_3_H_7_^+^ or C_2_H_3_O^+^ ions. A PEPIPICO map (not shown) reveals plenty of coincidences between the 6300 ns peak and CH_3_^+^, but hardly any with O^+^ or OH^+^, thus favoring the attribution of the 6300 ns peak to C_2_H_3_O^+^.

**Figure 4:**
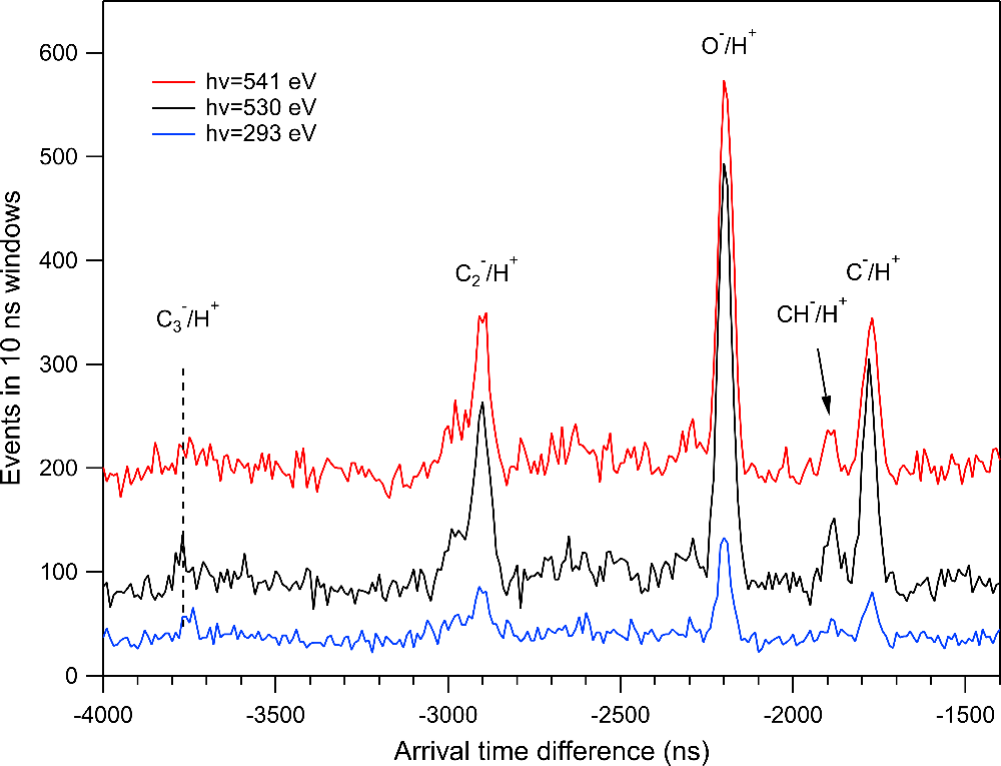
NIPICO spectra measured at photon energies 293, 530, and 541 eV (from bottom to top). These photon energies can induce photoionization but not resonant photoexcitation. The 293 eV spectrum was collected for 2 h, and the other two spectra for 4 h. The spectra have been moved vertically for clarity.

For comparison with the *resonant* measurements at the O 1s edge, in Figure 4 we show NIPICO spectra collected upon *nonresonant* core-level photoionization, using the photon energies 293, 530, and 541 eV. The lowest photon energy corresponds to photoionization just above the two C 1s ionization potentials (290.7 and 292.2 eV^19^), but it can also induce valence photoionization processes. The 530 eV energy is below the O 1s excitations, leading to dissociation events mainly from C 1s photoionization and valence photoionization channels. At 541 eV, events coming from O 1s single-hole photoionization are added to the just mentioned assembly of ionization processes. Despite the low statistics of the spectra, the same negative ions are apparently produced in the core photoionization continua as at the O 1s core excitations. Anion formation is expected to be enhanced also at C 1s core excitations, although reliable spectra for them could not be measured.

Due to normalization problems discussed above, we cannot quantify the increase in anion formation at the O 1s excitations of 2-propanol. However, such an increase was observed in our previous studies, e.g.^13,22,23^ To explain the mechanism of anion formation, we assume that 2-propanol is not an exemption. Core-excited molecules mostly decay by either (Auger) electron or (fluorescence) photon emission (ultrafast dissociation before core-hole decay^24^ is not considered here). For holes in the C 1s and O 1s orbitals, Auger electron emission is much more probable. In resonant core excitation, the electron promoted to an empty orbital can act as a spectator or participate in the decay process, so that either a 2-hole 1-particle state or a 1-hole state is respectively produced. The 2-hole 1-particle final states of spectator decay are often dissociative. Due to charge conservation, mostly neutral and singly charged positive fragments are created in dissociation after spectator decay. However, it appears that anions can also be formed after spectator decay in many molecules. If an anion is formed, it should be accompanied by two singly charged cations or by one doubly charged cation to conserve the total charge in the reaction. It is currently unknown exactly which kind of final states favor the formation of anions. Note that although our measurement scheme is selective to the excitation (photon) energies and to the detected final ionic products, it is not explicitly selective to the states and electronic transitions involved in the mediating dynamics that ultimately lead to anion production. Anion formation can also take place after some final states of normal Auger decay dissociate.

The binding energy range of the spectator final states can in principle be obtained from measuring the resonant Auger electron spectrum; however, to our knowledge, such spectra have not been published for 2-propanol. From the spectra of other oxygen-containing molecules,^25^ we estimate that the spectator final states are in the binding energy range of 20–70 eV. If some spectator final states have higher binding energies than the dissociation energy of a given fragmentation channel, the corresponding dissociation is energetically allowed. Unfortunately, the dissociation energies and the appearance energies of the most ions observed in this study are unavailable for 2-propanol. Their calculations are beyond the scope of the present study. However, we note that bond dissociation energies in 2-propanol and other small alcohols are of the order of 100 kcal/mol (or 4.3 eV).^26^ Considering the appearance of the C_3_^–^ ion in our experiments, there should be sufficient energy in the molecular cation after some spectator transitions for breaking all bonds of the molecule except for the C_3_ skeleton, and for ionizing two atomic fragments (H^+^ or O^+^).

### III.3 NIPIPICO Spectra

**Figure 5:**
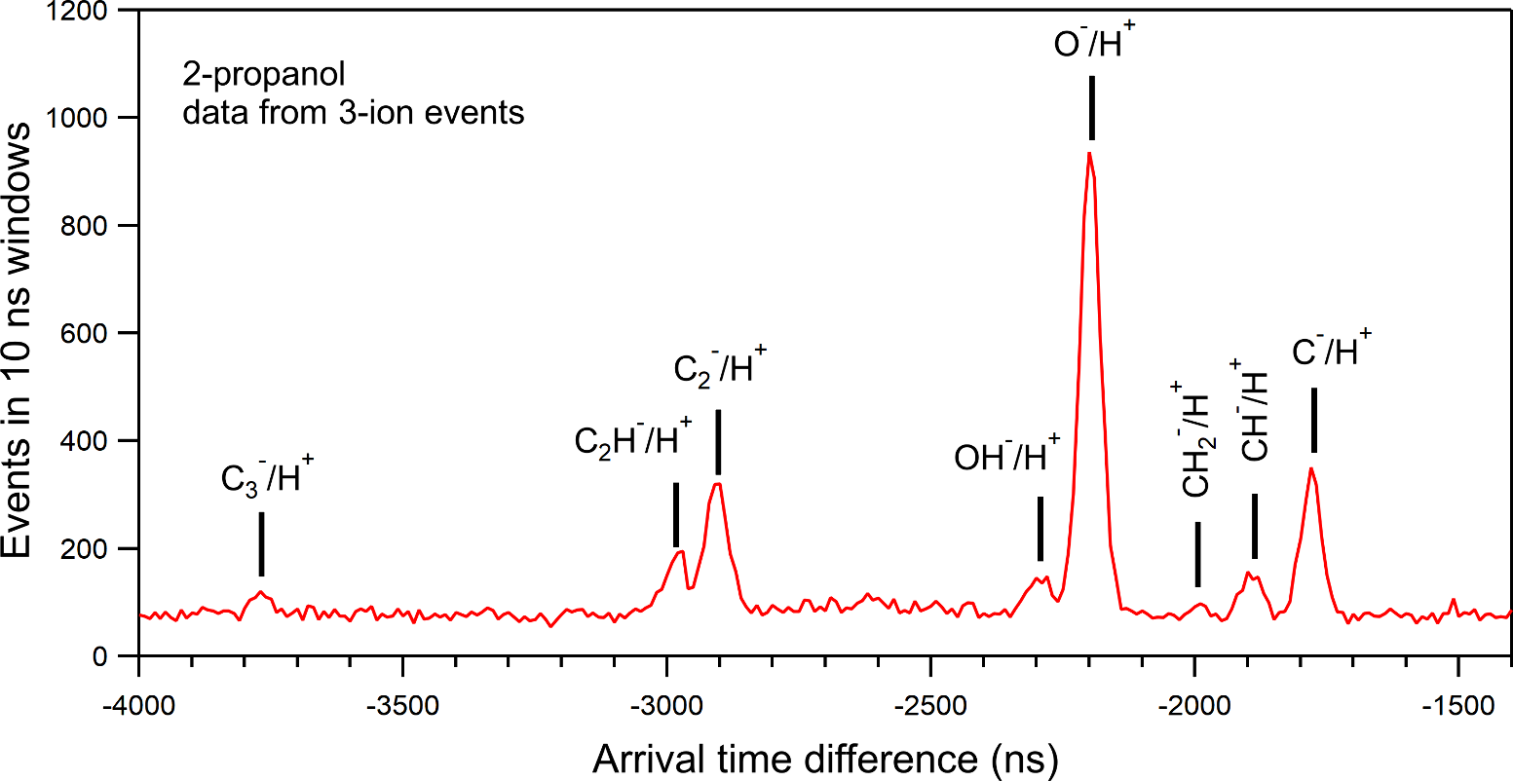
Part of the ATD spectrum for NI/PI_1_ coincidences extracted from three-ion coincidence events. For better statistics, the data collected using the three different photon energies below the O 1s IP (labeled A, B, and C in Figure 1) were summed together. The vertical bars indicate the calculated arrival time differences between the labeled negative and positive ions originating from the same parent molecule.

**Figure 6:**
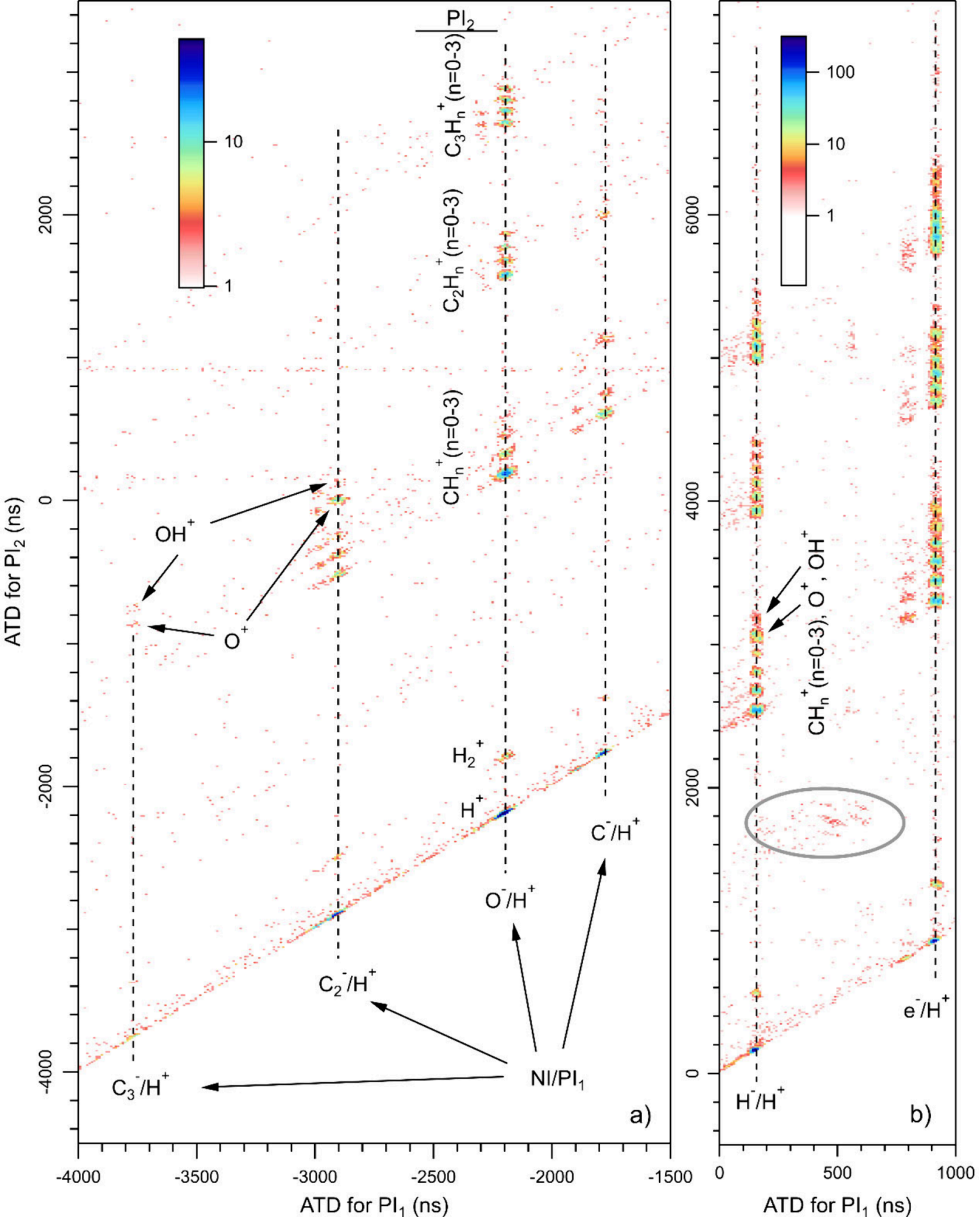
Negative-ion/positive-ion/positive-ion coincidence maps of 2-propanol. For better statistics, the data collected using the three different photon energies below the O 1s IP (labeled “A”, “B”, and “C” in Figure 1) are summed together. The maps were constructed by performing two-dimensional binning in 10 ns × 10 ns squares. The intensity scale is logarithmic with the dark blue color showing the highest intensity, and different for panels (a) and (b).

To increase the statistics for weak coincidence events, the data taken at the three different photon energies indicated by the arrows in Figure 1 were summed together. Coincidence events involving three or more particles were also registered in these measurements. Figure 5 shows the ATD spectrum between the negative ion and the first positive ion extracted from NI/PI_1_/PI_2_ coincidence events. Using this subset of data enhanced the signal-to-noise ratio, compared to two-ion coincidences NI/PI. The vertical bars show the expected positions of the NI/H^+^ coincidence peaks, and they match very well with the observed peak positions (the H^–^/H^+^ and O^–^/H^+^ peaks were used for calibration). In addition to the eight different anions mentioned above, the spectrum in Figure 5 suggests the presence of the CH_2_^–^ anion. The C^–^/H^+^ peak may have a minor contribution from O^–^/H_2_^+^ coincidences that are calculated to have very similar ATD (see also Figure 6).

Figure 6 shows two parts of a coincidence map for NI/PI_1_/PI_2_ events, where one negative ion and two positive ions were detected in coincidence. This image resulted from binning coincidence events in 10 ns × 10 ns squares along the two axes (ATD of PI_1_ and ATD of PI_2_). Coincidence events involving the three ions form islands or distinct patterns on the map. In Figure 6a, the first positive ion, PI_1_, of the NI/PI_1_/PI_2_ coincidences is always H^+^. Three-ion coincidences align vertically if the negative ion and the first positive ion (H^+^) are the same and the second positive ion (PI_2_) varies. To guide the eye, vertical dashed lines indicate the positions where H^–^/H^+^/PI_2_, C^–^/H^+^/PI_2_, O^–^/H^+^/PI_2_, C_2_^–^/H^+^/PI_2_, and C_3_^–^/H^+^/PI_2_ coincidences are expected to appear. The coincidence islands with the lightest PI_2_ are located at the smallest ATD values. The second positive ion (PI_2_) of the coincidences is labeled for some species on the left side of the vertical line for O^–^/H^+^. The assignment of PI_2_ for the other vertical lines is analogous, noting that all coincidence islands for a given PI_2_ align diagonally in Figure 6. For example, the lowest island in each vertical line originates from NI/H^+^/H^+^ coincidences.

For all the observed anions, the most common three-ion events involve two protons, i.e., NI/H^+^/H^+^ coincidences, and the most intense channel of all is H^–^/H^+^/H^+^. The H^–^/H^+^ ion pair is observed in coincidence with many different PI_2_ ions, among which the second most likely, after H^+^, is C^+^. Remarkably, the C_3_H_*n*_^+^ (*n* = 0–3) ions observed with the H^–^/H^+^ ion pair have slightly higher intensities than the C_2_H_*n*_^+^ (*n* = 0–3) ions. In Figure 6b, the ATDs for these PI_2_ ions are located around 5000 ns for C_3_H_*n*_^+^ (*n* = 0–3) and around 4000 ns for C_2_H_*n*_^+^ (*n* = 0–3). PI_2_ ions with the largest sizes are either very weak or completely missing. For example, clear signs of the H^–^/H^+^/C_2_OH_*n*_^+^ (*n* > 3) and H^–^/H^+^/C_3_OH_n_^+^ (any *n*) coincidences are not observed.

As shown in Figure 6a, the O^–^/H^+^ ion pair is also observed in coincidence with many different positive ions (including H_2_^+^), but only weakly with CH_3_^+^. The most likely PI_2_ other than H^+^ is again C^+^. The C_3_H_*n*_^+^ (*n* = 0–3) ions observed with the O^–^/H^+^ ion pair have similar intensities to the C_2_H_*n*_^+^ (*n* = 0–3) ions. The C^–^/H^+^ ion pair is mainly observed with H^+^, C^+^, CH^+^, O^+^, and C_2_^+^. The C_2_^–^/H^+^ ion pair appears with H^+^, H_2_^+^, C^+^, CH^+^, CH_2_^+^, and O^+^. Three-ion coincidences involving the C_3_^–^/H^+^ ion pair are all rather or very weak, but distinguishable in the C_3_^–^/H^+^/H^+^, C_3_^–^/H^+^/O^+^, and C_3_^–^/H^+^/OH^+^ dissociation channels. The observation of three-ion coincidences at expected positions provides additional support for the formation of the concerned anions. The weakest anion in Figure 5, CH_2_^–^, remains elusive in the NIPIPICO map of Figure 6a. It appears to show some coincidences on the bottom diagonal, i.e., CH_2_^–^/H^+^/H^+^ events, at the correct position (−1994 ns), but not much else. Figure 6b also shows weak traces of three-ion coincidences for which PI_1_ was not H^+^, but a heavier cation. Their positions are indicated by a gray ellipse, and they can be assigned to O^–^/CH_n_^+^(n = 0–3)/C_2_H_n_^+^(n = 0–3) coincidences. The most intense channel among them is O^–^/CH_2_^+^/C_2_H_2_^+^.

Some of the coincidence signals cannot be explained by any conceivable correctly measured photodissociation processes. These are marked with the symbol ”?” in Figure 2, and are visible also in the NIPIPICO map in Figure 6 at corresponding ATDs. They do, however, seem to be related to real coincidence events, and, for example, the signals at +50 ns and at +800 ns are likely related to their adjacent real coincidence signals, as labeled in Figure 6b. The lower values of these ATDs imply either too early stop signals from the positive ion detector or delayed start signals from the negative particle detector. The latter seems more likely based on the NIPIPICO maps and could be caused by triggers from secondary particles and/or indirect flight trajectories due to hits to the meshes or spectrometer walls, with electron trajectories additionally being perturbated by the magnetic field. Notice that in Figure 2 the relative intensity of the peak at around +800 ns, which is presumably related to e^–^/H^+^ coincidences at 920 ns, decreased considerably when some magnets were removed upon going from the NIPICO settings to the PEPICO settings. A relevant question is whether contaminating coincidence islands at ATDs just below intense NIPICO channels are present for all real coincidence events and could lead to misinterpretation of some the assigned features. Namely, such contaminating events could contribute to the intensities of the minor anions CH^–^, CH_2_^–^, OH^–^, and C_2_H^–^. However, based on the variations in the relative intensities of the different peaks, we think that the major contributions for these peaks do come from real coincidences. Additionally, Figure 2 shows that the unknown coincidence peaks are wider than the real NI/PI coincidence peaks such as H^–^/H^+^, whereas in Figure 5 all the assigned NI/H^+^ peaks have comparable widths; none of them appear suspiciously wide.

In Figure 6, coincidence islands display quite different slopes, both positive and negative. The analysis of the slopes could, at least in principle, reveal information on how fragmentation takes place. However, we omit the discussion about possible explanations (such as concerted and sequential dissociation) in this work because its nature would be too speculative.

## IV Conclusions

The soft X-ray induced production of negative ions from gas-phase 2-propanol molecules was studied at the O 1s edge using negative-ion/positive-ion coincidence spectroscopy. The formation of a large variety of anions—nine in total (H^–^, C^–^, CH^–^, CH_2_^–^, O^–^, OH^–^, C_2_^–^, C_2_H^–^, and C_3_^–^)—was observed. Additionally, we found that negative ions were produced in nonresonant photoionization at photon energies above the C 1s and O 1s edges. At all photon energies used, the most abundant anionic fragment was found to be H^–^, followed by O^–^. The analysis of three-ion coincidence (NIPIPICO) events proved highly advantageous for the assignment of the spectral signatures. For all the observed anions, the most common three-ion events were those involving two protons (NI/H^+^/H^+^).

It is apparent that the diversity of anions produced in X-ray excitation processes increases with the complexity of the system, and further experiments on large polyatomic molecules are warranted. Future studies may also focus on performing more detailed analyses of the fragmentation pathways via, e.g., slope analysis of the coincidence islands and momentum analysis of the ionic fragments.^11^ Explicit information on the electronic states and decay channels mediating anion production could be obtained by including also the measurement of electron kinetic energies in the coincidence scheme.
